# Renal Infarction in a Young Man

**DOI:** 10.4274/balkanmedj.galenos.2019.2019.1.73

**Published:** 2019-08-22

**Authors:** Zehra Eren, Hakan Koyuncu

**Affiliations:** 1 Department of Nephrology, Alanya Alaaddin Keykubat University School of Medicine, Antalya, Turkey; 2 Department of Urology, Yeditepe University School of Medicine, İstanbul, Turkey

A 43-year-old man presented to the emergency department with a 1-h history of severe abdominal pain, particularly localized in the umbilical area and accompanied by cold sweating. His medical history was unremarkable. Abdominal examination revealed no defense or rebound tenderness (1). Vital signs as well as systemic and laboratory findings were normal, except for the elevated levels of aspartate aminotransferase (120 U/L; reference range, <46 U/L) and lactate dehydrogenase (977 U/L; reference range, 135-225 U/L), indicating signs of damage in the body tissues. Abdominal ultrasonography revealed normal findings, and doppler ultrasonography revealed no flow in the left inferior segmental renal artery. Furthermore, abdominal computed tomography with contrast revealed a hypodense area in the left kidney involving the anterolateral component of the upper and middle zones in addition to the entire lower pole (Figure 1). Selective renal angiography performed to rule out pathologies that require interventions (stenosis of a large artery, dissection) demonstrated 80% stenosis in the middle inferior segmental renal artery (Figure 2). A diagnosis of renal infarction was made, and treatment with enoxaparin (2×0.6 mL/day) was initiated (2). The patient’s pain resolved within the next 24 h; however, the levels of aspartate aminotransferase and lactate dehydrogenase continued to increase. Clopidogrel (75 mg/day) and aspirin (100 mg/day) were added to the treatment. The elevated enzyme levels began to decrease after 48 h of presentation, and the renal functions remained normal. Further investigation to determine the etiology of the infarction (presence of malignancy and hematological problems) revealed normal results; therefore, we determined this case as idiopathic. Although the patient’s renal function remained normal, 99mTc-dimercaptosuccinic acid renal scintigraphy performed 1 month later revealed cortical defects in the upper and lower lateral poles of the left kidney (Figure 3). Written informed consent was obtained from the patient.

## Figures and Tables

**Figure 1:**
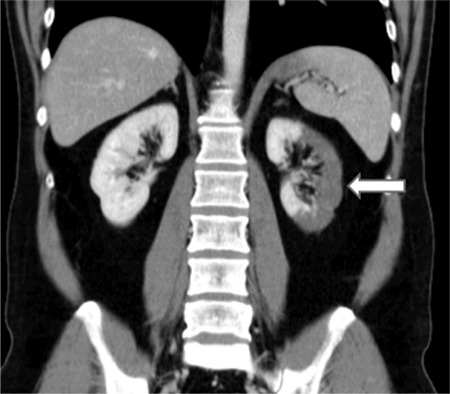
Abdominal computed tomography with contrast showing a hypodense area in the left kidney involving the anterolateral component of the upper and middle zones and the entire lower pole.

**Figure 2:**
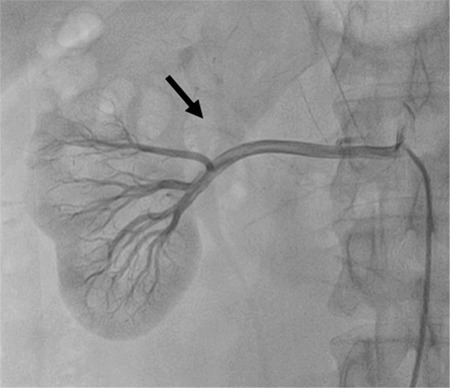
Selective renal angiography showing 80% stenosis in the middle inferior segmental renal artery.

**Figure 3:**
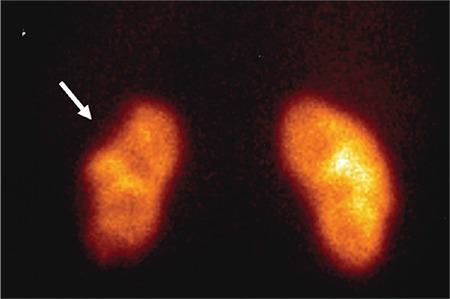
99mTc-dimercaptosuccinic acid renal scintigraphy showing cortical defects in the upper and lower lateral poles of the left kidney (anterior position).
